# The Effect of Local Injections of Bupivacaine Plus Ketamine, Bupivacaine Alone, and Placebo on Reducing Postoperative Anal Fistula Pain: A Randomized Clinical Trial

**DOI:** 10.1155/2014/424152

**Published:** 2014-12-03

**Authors:** Alireza Kazemeini, Mojgan Rahimi, Mohammad Sadegh Fazeli, Seyedeh Adeleh Mirjafari, Hamid Ghaderi, Kamal Fani, Mohammad Forozeshfard, Marzieh Matin

**Affiliations:** ^1^Department of General Surgery, Imam Khomeini Hospital Complex, Tehran University of Medical Sciences, Tehran, Iran; ^2^Department of Anesthesiology, Imam Khomeini Hospital Complex, Tehran University of Medical Sciences, Tehran, Iran; ^3^Brain and Spinal Injury Research Center (BASIR), Tehran University of Medical Sciences, Tehran, Iran; ^4^Department of Cardiovascular Surgery, Shahid Modarres Hospital, Shahid Beheshti University of Medical Sciences, Tehran, Iran; ^5^Department of Anesthesiology, Shahid Modarres Hospital, Shahid Beheshti University of Medical Sciences, Tehran, Iran; ^6^Department of Anesthesiology, Semnan Medical University, Semnan, Iran

## Abstract

*Background and Objective*. This study aimed to compare the effects of different local anesthetic solutions on postoperative pain of anal surgery in adult patients. *Method*. In this randomized double-blind prospective clinical trial, 60 adult patients (18 to 60 years old) with physical status class I and class II that had been brought to a university hospital operating room for fistula anal surgery with spinal anesthesia were selected. Patients were randomly divided into 4 equal groups according to table of random numbers (created by Random Allocation Software 1). Group 1 received 3 mL of normal saline, group 2, 1 mL of normal saline plus 2 mL of bupivacaine 0.5%, group 3, 1 mL of ketamine plus 2 mL of bupivacaine 0.5%, and group 4, no infiltration. Intensity of pain in patients was measured using visual analogue scale (VAS) at 0 (transfer to ward), 2, 6, 12, and 24 hours after surgery. Time interval to administration of drugs and overall dose of drugs were measured in 4 groups. *Results*. Mean level of pain was the lowest in group 3 at all occasions with a significant difference, followed by groups 2, 4, and lastly 1 (*P* < 0.001). Furthermore, groups 2 and 3 compared to groups 1 and 4 had the least overall dose of analgesics and requested them the latest, with a significant difference (*P* < 0.05). *Conclusion*. Local anesthesia (1 mL of ketamine plus 2 mL of bupivacaine 0.5% or 1 mL of normal saline plus 2 mL of bupivacaine 0.5%) combined with spinal anesthesia reduces postoperative pain and leads to greater comfort in recovering patients.

## 1. Introduction

Anorectal diseases affect nearly 5% of the adult population [1]. Anal fistula is a common anorectal problem and complaint of more than 10% of visits to anorectal clinics [2]. Fistula surgery can be performed under general, spinal, or local anesthesia [3].

Anal surgery is usually performed as inpatient, and the main reason for this is the concern about lack of postoperative pain control and associated problems [2]. Today, various medications have been studied for postoperative analgesia as suppositories, local anesthesia, or oral preparations. Yet the search for a suitable combination still continues.

Although general anesthesia, spinal anesthesia, and nerve blocks [1, 4–9] have been examined in anorectal surgeries, combined local and spinal anesthesia for greater patient comfort after anorectal surgery and pain control has been studied less.

No comparison has yet been carried out on analgesic effect of local injection of bupivacaine or bupivacaine plus ketamine. Several studies have addressed additional injection of local ketamine to local anesthetics in surgeries such as adenotonsillectomy [10], herniorrhaphy [11], and intra-articular injection [12]. Given the above, it appears that peripheral use of ketamine is an ideal postoperative pain reduction technique.

This study was conducted with the aim to assess and compare analgesic effects of local bupivacaine and bupivacaine plus ketamine with placebo and no-intervention groups in patients undergoing anal fistula surgery. Possible side effects of these drugs were also studied.

## 2. Materials and Methods 

This randomized double-blind prospective clinical trial was conducted on 60 adult patients (18- to 60-year-old) with physical status class I and class II, candidate for no-complicated fistula repair surgery under spinal anesthesia in Imam Khomeini Hospital over one year (2010-2011). Study inclusion and exclusion criteria are shown in Table 1. All patients were briefed about surgery and study procedures, and if they wished, they were invited to participate. Informed consent for participation in study was obtained from every patient, and scientific, practical, and ethical integrity of study was approved by the Surgery Department of Tehran University of Medical Sciences. This study was performed in accordance with principles of Helsinki Declaration and checklist of ethics in research.

Sampling was conducted using convenient sampling method. In this study, a sample size of 15 patients per group was studied. Patients were randomly divided into 4 groups using Random Allocation Software 1.

All patients underwent spinal anesthesia in sitting position at L3-L4 with hyperbaric injection of 1 mL of lidocaine 5% (Quincke needle number 25). After 1.5 minutes in sitting position and ensuring appropriate level of anesthesia, patients were placed in supine position and then into lithotomy position, and fistula repair surgery was performed by the same surgeon. After surgery, syringes containing above-mentioned drugs (all looked the same and had clear contents) were handed to the surgeon for injection at above-mentioned dentate line.

Only the principle researcher had a copy of the table containing patients' information in each group. This information was kept confidential until the end of the study. Considering that patients were under anesthesia and sedation, they did not know of injection or otherwise of any drugs. Moreover, since drugs were used in the operating room, neither the patient nor follow-up person had any information about the type of drug used. Thus, neither for the patient nor for the person collecting data was it possible to recognize patient's group.

Content of syringes was according to grouping including group 1 (3 mL of normal saline), group 2 (1 mL of normal saline plus 2 mL of bupivacaine 0.5%), group 3 (1 mL of ketamine plus 2 mL of bupivacaine 0.5%), and group 4 (no infiltration), and the following variables were measured in each group: age, gender, weight, height, and intensity of postoperative pain using VAS, on occasions of transfer to ward and 2, 6, 12, and 24 hours after transfer. Time intervals of the first request for analgesic, overall dose of analgesic, nausea and vomiting, receiving anti-vomiting and nausea drug (metoclopramide 10 mg/kg IV) according to patient's request, or nausea and vomiting more than 10 minutes, and psychological complications (hallucination, delirium) were also measured. In VAS, the intensity of pain is shown linearly from 0 to 10, and patient is trained to express level of pain from 0 (no pain) to 10 (most pain ever experienced by the patient).

A questionnaire was prepared for each patient, which was completed using an interview and examination results and patient history. Data were analyzed with SPSS-16, and descriptive data were presented in the tables and figures. *P* < 0.05 was considered significant.

In the event of pain (VAS > 3) or patient request for analgesic, 25 mg of intramuscular pethidine was administered, and thus patients received the best possible treatment (without interfering with their current treatment) and did not have to bear any extra pain because of this study.

In the present study, it was unlikely to miss patients, given short duration of the study and its completion in the operating room and ward. All patients were followed up until healing of their wound.

## 3. Results

All results and variables studied are presented in Table 2.

Sixty patients with a mean age of 34.87 ± 8.73 years and age range of 20 to 60 years were studied over 12 months, of whom 41 (68.3%) were male and 19 (31.7%) were female. There were insignificant differences between the 4 groups in terms of age distribution (*P* = 0.123, using ANOVA test), gender (*P* = 0.928, using Chi-square test), mean height (*P* = 0.999), mean weight (*P* = 0.795), or mean BMI (*P* = 0.347).

Among patients, mean time of the first opiate injection was 5.93 ± 3.209 hours, with a range of 0 to 17 hours, which is presented separately for each group in Table 2, with a significant difference between groups (*P* = 0.015 using ANOVA test), such that groups 2 and 3 were last to request analgesics. Furthermore, mean overall dose of opiate used was 6.17 ± 3.836 mg, ranging from 0 to 15 mg, with significant differences between groups (*P* < 0.001, using ANOVA), also shown in Table 2, such that groups 2 and 3 had the least need for opiates (pethidine).

**Figure 1:**
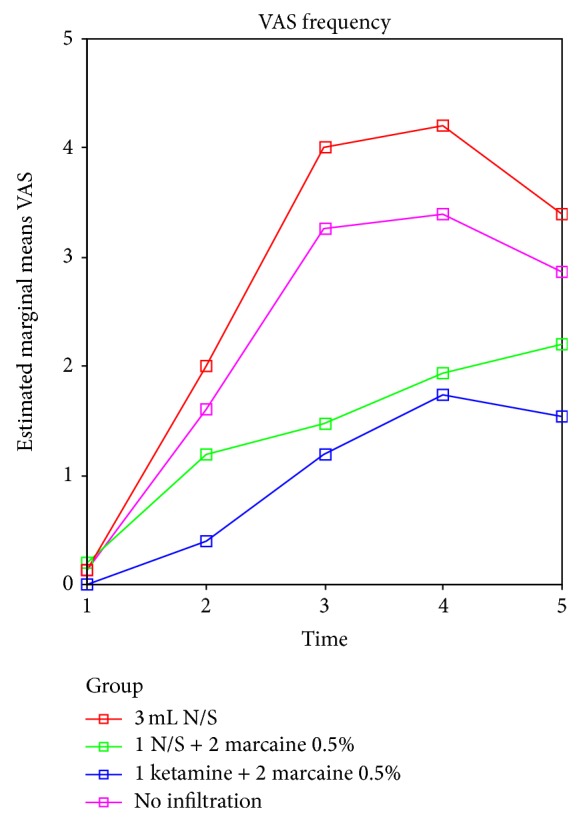
Level of pain at different times after surgery for separate groups. ANOVA test was used to compare groups. There was a significant difference in pain intensity between groups. Level of pain significantly increased and then decreased. There was an insignificant difference in pain intensity between groups 2 and 3. However, there was a significant difference between groups 2 and 3 and 1 and 4 (*P* < 0.001). Group 1 (3 mL of normal saline), group 2 (1 mL of normal saline plus 2 mL of bupivacaine 0.5%), group 3 (1 mL of ketamine plus 2 mL of bupivacaine 0.5%), and group 4 (no infiltration).

There were significant differences between groups in pain assessment times (*P* < 0.001, using ANOVA), as shown in Table 3 and Figure 1, for different groups and assessment times.

In VAS method, mean pain in group 3 (1 mL of ketamine plus 2 mL of bupivacaine 0.5%) was the lowest at all times, so that, at the time of transfer to ward, mean pain was 0 ± 0.00, and 2 hours later it was 0.4 ± 0.507, 6 hours later 1.2 ± 0.561, 12 hours later 1.73 ± 0.594, and 24 hours later 1.53 ± 0.64, which was the lowest on all occasions compared to other groups, followed by group 2. The highest level of pain was measured in group 1.

Of the 60 patients, 14 (23.3%) required bladder catheterization (*P* = 0.054, using Chi-square test), 16 had nausea and vomiting (*P* = 0.977, using Chi-square), and 7 required administration of 10 mg of metoclopramide (*P* = 0.82, using Chi-square test), with insignificant differences between 4 groups. No psychiatric problems (hallucination and delirium) were observed among any of the 60 patients.

## 4. Discussion

There are only a few studies on rectal administration of analgesics following anal surgeries [13]. There are no similar studies so far, to compare analgesic effect of local injection of bupivacaine or bupivacaine with ketamine in postrectal surgeries. The present study results showed significant and favorable analgesic effect of local injection of bupivacaine with ketamine, followed by analgesic effect of bupivacaine alone.

Two pain reduction techniques in anorectal surgeries include local infiltration and block [9, 14, 15]. A variety of different choices of local anesthetics have been proposed, which include lidocaine [16, 17], ropivacaine [4, 18, 19], or a combination of lidocaine and bupivacaine [1, 5, 8, 17, 20] with [1, 5, 8, 17, 20, 21] or without adrenaline [4, 16, 18, 19].

Many studies have so far investigated the effect of adding local ketamine injection to local anesthetics; for instance, Dal et al. [10] showed that local injection of ketamine in children undergoing adenotonsillectomy significantly reduced pain score, dose of rescue analgesia, and increased time interval to the first dose of opiate compared to the group receiving IV normal saline. Tverskoy et al. [11] used local injection of 0.5 mg/kg ketamine after herniorrhaphy to reduce pain and showed that ketamine improved the quality of anesthesia and analgesia created by local anesthetics used in these patients.

Given these results, it appears that peripheral use of ketamine is an ideal postoperative pain reduction technique.

Both Jack knife [4, 8, 16, 18, 19] and lithotomy [5, 17] positions have been proposed for anorectal surgeries. In the present study, lithotomy position was preferred, so that, in case of ineffective spinal anesthesia, general anesthesia could be applied without changing position, even though this was not required, and anesthesia was complete in every case, and no surgery had to be terminated, nor did local anesthesia change to general anesthesia in any patient, which concurs with other studies [22–24].

In the present study, mean age was 34.87 ± 8.73 years (range of 20 to 60 years), but, in other studies, age ranged from 35 to 45 years [21, 25, 26]. In the present study, there was no significant difference between the groups in terms of age (*P* = 0.123), and mean age was similar to other studies.

In various studies, there was a wide range in terms of prevalence in different sexes. In studies by Oh et al. [27] and Melange et al. [25], 55.2% of patients were men, and in studies by Nahas et al. [26] 84% were men. In the present study, there was no significant difference between the 4 groups in terms of gender distribution (using Chi-square test) (*P* = 0.928), which is similar to other studies.

Mean time of the first injection of opiates among patients was 5.93 ± 3.209 hours, ranging from 0 to 17 hours, with a significant difference between groups (*P* = 0.015, using ANOVA test), so that group 1 (normal saline injection) with a mean of 4.4 hours was the first to request opiates, and group 3 (1 mL of ketamine plus 2 mL of bupivacaine 0.5%) with a mean of 7.2 hours and group 2 (1 mL of normal saline plus 2 mL of bupivacaine 0.5%) with a mean of 7.27 hours were last. No study was found in this regard. The mean overall dose of opiates required by patients was 6.17 ± 3.836 mg, ranging from 0 to 15 mg, with a significant difference between groups (*P* < 0.001, using ANOVA), such that patients in group 1 with an overall mean dose of 9.5 mg received the highest dose of opiates and group 3 with an overall mean dose of 2.33 mg, the lowest. According to ANOVA test, there was a significant difference between groups (*P* < 0.001, using ANOVA), and, according to analgesic measurement, this level was sufficient.

Level of postoperative pain according to VAS was between 1 and 4, which agreed with most of the other studies [4, 5, 17, 18]. Mean pain level at all times was the lowest in group 3, followed by groups 2, 4, and 1, respectively. Groups 1 and 4 had the highest level of pain. These results are in line with studies that used general, spinal, or local anesthesia to reduce pain, including studies by Place et al. [28], Notaras [29], and Bell [30].

In terms of complications, no cases of hematoma, bleeding, or infection were observed. In terms of urinary retention, 23% required bladder catheterization, and the difference between groups was insignificant. Urinary retention, following anorectal surgery, was reported between 7% and 20% [5, 17, 31]. Although urinary retention has been reported negligible following perianal block for pain reduction and faster ambulation of patient [21], this was not observed in the present study.

Patients were discharged with NSAID medication, and their pain gradually diminished in 1 to 3 days, which was similar to results in other studies [4, 17, 21].

## 5. Conclusion

According to the present study results, local anesthesia (1 mL ketamine plus 2 mL bupivacaine 0.5%, or 1 mL normal saline plus 2 mL bupivacaine 0.5%) in combination with spinal anesthesia leads to reduced postoperative pain and greater patient comfort during recovery.

## Figures and Tables

**Table 1:** Study inclusion and exclusion criteria.

Exclusion criteria	Inclusion criteria
Patients with contraindications for spinal anesthesia (refusal, infection at puncture site, and history of coagulation problems, etc.)	Noncomplicated and singular fistula

Patients with a history of daily intake of NSAID	All participating patients were in physical class I and class II

Patients with a history of rectal or anal surgery	Aged 18 years to 60 years

Other concomitant diseases in anal or rectal area	Consent to surgery under spinal anesthesia

Contraindications to use of study drug (allergy to local anesthesia such as a history of methemoglobinemia, erythema, urticaria, allergic dermatitis, hyperpigmentation and purpura after ingestion, mental diseases, asthma, hypertension, congenital and acquired heart diseases, etc.)	

Drug addicts or patients with a history of alcohol or drug use	

Pregnant and lactating patients	

Need to change local to general anesthesia	

BMI > 30 kg/m^2^	

**Table 2:** Studied variables.

	Group 1	Group 2	Group 3	Group 4	Total	Test	*P*
Age	33.87 ± 7.43	38.93 ± 7.34	31.47 ± 6.51	35.20 ± 11.78	34.87 ± 8.73	ANOVA	*P* = 0.123
Sex (F/M)	6/9	4/11	4/11	5/10	19/41	Chi-square test	*P* = 0.928
High (cm)	165.60 ± 8.62	165.07 ± 8.68	165.53 ± 9.66	165.33 ± 9.68	165.38 ± 9.00	ANOVA	*P* = 0.999
Weight (kg)	71.87 ± 7.47	73.47 ± 9.99	74.07 ± 9.24	71.13 ± 9.03	72.63 ± 8.88	ANOVA	*P* = 0.795
BMI	26.20 ± 2.07	26.82 ± 1.76	26.92 ± 1.31	25.94 ± 1.73	26.47 ± 1.47	ANOVA	*P* = 0.347
Time of operation	14.3 ± 2.669	12.47 ± 2.031	13.40 ± 2.098	13.80 ± 2.145	13.05 ± 2.507	ANOVA	*P* = 0.06
First administration of opiate (hour)	4.40 ± 1.05	7.27 ± 4.09	7.20 ± 4.04	4.87 ± 1.35	5.93 ± 3.20	ANOVA	*P* = 0.015
Total administration of opiate (pethidine mg)	9.50 ± 2.86	4.67 ± 2.47	2.33 ± 1.48	8.17 ± 3.33	6.17 ± 3.83	ANOVA	*P* = 0.000
Need for bladder catheterization (*n*)	4	5	0	5	14	Chi-square test	*P* = 0.054
First urination (hour)	6.00 ± 2.45	5.93 ± 1.10	5.07 ± 1.33	5.93 ± 1.33	5.48 ± 1.79	ANOVA	*P* = 0.06
Nausea and vomiting (*n*)	4	4	5	3	16	Chi-square test	*P* = 0.977
Need for administration of 10 mg metoclopramide (*n*)	1	3	2	1	7	Chi-square test	*P* = 0.820
Score sedation (awake/drowsy/somnolent)	11/1/3	12/1/2	10/1/4	12/1/2	45/4/11	ANOVA	*P* = 0.988

**Table 3:** VAS frequency among groups.

VAS test	Group	Mean
VAS at transfer to ward	Group 1	0.13 ± 0.352
	Group 2	0.20 ± 0.414
	Group 3	0 ± 0.000
	Group 4	0.13 ± 0.516
	Total	0.12 ± 0.372

VAS 2 hours later	Group 1	2.0 ± 0.655
	Group 2	1.20 ± 0.414
	Group 3	0.40 ± 0.507
	Group 4	1.60 ± 0.507
	Total	1.30 ± 0.788

VAS 6 hours later	Group 1	4.0 ± 0.926
	Group 2	1.47 ± 0.516
	Group 3	1.2 ± 0.561
	Group 4	3.27 ± 0.458
	Total	2.48 ± 1.347

VAS 12 hours later	Group 1	4.2 ± 0.561
	Group 2	1.93 ± 0.458
	Group 3	1.73 ± 0.594
	Group 4	3.40 ± 0.507
	Total	2.82 ± 1.157

VAS 24 hours later	Group 1	3.4 ± 0.986
	Group 2	2.2 ± 0.561
	Group 3	1.53 ± 0.640
	Group 4	2.87 ± 0.352
	Total	2.5 ± 0.966

ANOVA test	*P* < 0.001	
